# Optical-Based Artificial Palpation Sensors for Lesion Characterization

**DOI:** 10.3390/s130811097

**Published:** 2013-08-21

**Authors:** Jong-Ha Lee, Yoon Nyun Kim, Jeonghun Ku, Hee-Jun Park

**Affiliations:** 1 Department of Biomedical Engineering, School of Medicine, Keimyung University, 1095, Dalgubeol-daero, Daegu 704-701, Korea; E-Mail: segeberg@kmu.ac.kr (J.-H.L.); Tel.: +82-53-580-3736; Fax: +82-53-580-3746; 2 Department of Internal Medicine, Dongsan Medical Center, Keimyung University, 1095, Dalgubeol-daero, Daegu 704-701, Korea; E-Mail: ynkim@dsmc.or.kr; Tel.: +82-53-580-3736; Fax: +82-53-250-7952

**Keywords:** tumor detection, artificial palpation, lesion characterization, optical sensor, tactile sensor, young's modulus

## Abstract

Palpation techniques are widely used in medical procedures to detect the presence of lumps or tumors in the soft breast tissues. Since these procedures are very subjective and depend on the skills of the physician, it is imperative to perform detailed a scientific study in order to develop more efficient medical sensors to measure and generate palpation parameters. In this research, we propose an optical-based, artificial palpation sensor for lesion characterization. This has been developed using a multilayer polydimethylsiloxane optical waveguide. Light was generated at the critical angle to reflect totally within the flexible and transparent waveguide. When a waveguide was compressed by an external force, its contact area would deform and cause the light to scatter. The scattered light was captured by a high-resolution camera and saved as an image format. To test the performance of the proposed system, we used a realistic tissue phantom with embedded hard inclusions. The experimental results show that the proposed sensor can detect inclusions and provide the relative value of size, depth, and Young's modulus of an inclusion.

## 1. Introduction

According to the American Cancer Society, more than 178,000 women and 2,000 men are diagnosed with breast cancer every year; international statistics report an estimated 1,152,161 new cases annually. This form of the disease is the leading killer of women between 40 and 55 years of age, and statistically, it is the second most prevalent cause of death overall in women. The common forms of breast cancer are lobular carcinoma *in situ*, ductal carcinoma *in situ*, infiltrating lobular carcinoma, and infiltrating ductal carcinoma; the rarer forms are medullary carcinoma, mucinous carcinoma, and tubular carcinoma. Extremely rare forms are inflammatory breast cancer and Paget's disease of the nipple—which account for only 1% of all breast cancers diagnosed—and Phyllodes tumors, which are fatal for less than 10 women annually in the United States. On a positive note is that the mortality rate in women with breast cancer is decreasing each year. This is due to the medical profession's emphasis on early detection methods, as well as emerging and more effective treatments. The ten-year survival rate is currently 98% of women who are diagnosed in the early stages of the disease (stage I); for cases where the cancer has progressed to stage III, the ten-year survival rate is 65%. Clearly, early detection and diagnosis is the key to surviving this often fatal disease. There are many methods used today to screen for various forms of breast cancer. A great deal of research is being conducted worldwide to develop new techniques for detection. The criteria for such modalities include accuracy, high sensitivity, acceptable specificity, ease of use, acceptability in terms of levels of discomfort and time taken to perform the test, and cost effectiveness.

Many researchers have studied noninvasive techniques for tumor detection over the past decade or so. This field has gained substantial impetus as computer-based imaging technologies have evolved and the computational power of present day systems is exponentially greater than in the past. The early detection of inclusions, tumors, and cancerous tissues is critical for the clinical cure of the disease. X-ray based techniques such as X-ray mammography are currently the most widely used and accepted methods worldwide. However, such techniques show low sensitivity in the presence of dense tissue and a high false-positive rate, as well as exposing the patients to radiation [1]. These shortcomings have led to the development of other techniques like ultrasonography, MRI, CT, PET, and infrared imaging. The thermal imaging technique, called thermography, is a noninvasive and efficient early detection method [2,3]. Recent enhancements in computerized thermograms, and the use of bio-statistical [4] and artificial intelligence [5,6] techniques have enhanced the reliability of tumor and breast cancer detection [6]. However, the complexities involved due to internal and external factors have not yet been fully quantified in various studies using thermal imaging techniques.

Another important technique involves the use of the elastic properties of inclusion and determination of their effect on the deformation and stress fields at the top surface of the tissue and bottom surface of the tactile sensor. In one of these techniques, researchers have focused on elastography to determine the tissue stiffness. This involves external excitation of a tissue and then measuring the resulting tissue motion [7–12]. To evaluate breast tumors via ultrasound, radiologists consider several features in the image, such as lesion shape, orientation, echo pattern, and posterior acoustic enhancement. Interpretation of ultrasound images, however, is subjective and variability is very high due to its low image resolution and the different experiences of radiologists who analyze the tumor features. Ultrasound was the first such technique used to measure tissue motion. The application of Doppler ultrasound to measure the tissue motion by harmonic excitation was first described by Parker and Lerner and is called sonoelasticity imaging [13]. These motions can also be transformed into strain readings by co-relating the uncompressed and compressed state of tissue [14]. This new approach of using tactile sensing to detect embedded objects is completely noninvasive. Dargahi and Najarian [15,16] conducted a comprehensive survey to review human tactile perception as a standard for this technology and evaluated advances in related fields and their impact on various applications. Hosseini *et al.* [17,18] proved the reliability and accuracy of these approaches for the detection of inclusions in biological tissues using Finite Element Modeling (FEM) Finally, Najarian *et al.* [19,20] proposed an analytical approach to predict the stiffness and geometric details of inclusions.

Recently, a new technological method such as fiber-based rolling indentation probe has been explored [21,22]. This type of technology calculates the stiffness distribution of a soft tissue while rolling over the tissue surface using fiber-based rolling indentation during minimally invasive surgery. The capacitive sensor based tactile imaging is also proposed in [23]. They measure the tissue stiffnesss using the capacitive coupling method. In [24], they analyses the force-sensing performance that would allow an instrumented kinesthetic probe to localize tumors based on stiffness variations of the lung parenchyma. The integrated approach for robotic palpation combined with biomechanical soft tissue characterization is proposed. However, the resolution of pressure sensor based these method is not as good as optically based method. Also the device requires other sensors to detect the applied force.

The tried and tested breast self-examination (BSE) is still recommended for the early detection of tumors, while a clinical breast examination (CBE) performed by a medical specialist still has a success rate of over 57% and specificity of more than 97%. Although these methods cannot determine the degree of malignancy, they do detect lesions that require further testing. The drawback of CBE is that the doctor performing the test is unable to fully describe what he or she feels, either orally or in writing. In this paper, we introduce an optical-based, artificial palpation sensor with a noninvasive, non-ionized, and easy-to-use breast cancer screening system. The palpation sensor is based on the fact that a tumor or malignancy feels different from the tissue surrounding it. When a doctor palpates the breast during CBE, he or she is able to feel a change in the composition of the tissue. Now, by way of modern technology, the palpation sensor takes this one step further in being able to quantitatively measure what is felt as an image that shows such parameters as its Young's modulus, the diameter of the tumor, and its depth. This is a cost-effective system; moreover, it is portable and requires minimal training of the operator. In the following section, the palpation sensor design concept is introduced. Then, the analytic solution and numerical simulation of the imaging principle are discussed. Next, the palpation sensor is validated using realistic tissue phantoms of different size, depth, and Young's modulus of inclusions. Finally, conclusions are presented.

## 2. Sensor Design and Sensing Principle

In this section, we present the design concept of the artificial palpation sensor in detail.

### 2.1. Sensor Design

**Figure 1.**
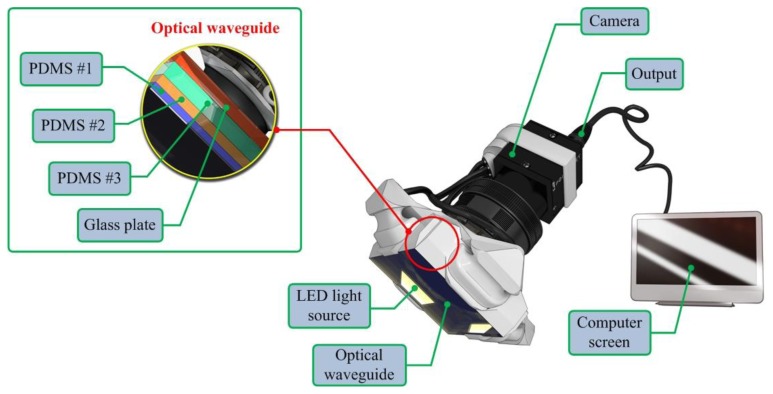
Schematic of the optical-based artificial palpation sensor.

The artificial palpation sensor incorporates an optical waveguide unit, a light source unit, a high-resolution camera unit, and a computer unit. The optical waveguide is the system's main sensing probe. The waveguide is composed of polydimethylsiloxane (PDMS), which is a high-performance silicone elastomer. In the current design, the waveguide needs to be flexible and transparent, and PDMS meets this requirement. To reach the level of sensation of human touch, we emulated the tissue structure of the human finger. The human finger tissue is composed of three layers with different elastic moduli, specifically the epidermis, dermis, and subcutaneous layer. The epidermis is the hardest layer, with the smallest elastic modulus, and it is approximately 1 mm thick. The dermis is a softer layer, and it is approximately 1 to 3 mm thick. The subcutaneous is the softest layer and fills the space between the dermis and bone. It is mainly composed of fat and functions as a cushion when a load is applied to the surface. Due to the difference in hardness of each layer, the inner layer deforms more than the outmost layer when the finger presses into an object. To emulate this structure, three PDMS layers with different elastic moduli were stacked together. PDMS layer 1 is the hardest layer, the PDMS layer 2 is the layer with medium hardness, and the PDMS layer 3 is the layer with the least hardness [9]. The height of each layer is approximately 2 mm for PDMS layer 1, 3 mm for PDMS layer 2, and 5 mm for PDMS layer 3. Figure 1 shows the schematic of the proposed sensor.

The high resolution camera was a mono-cooled complementary camera with an individual pixel size of 4.65 μm (H) × 4.65 μm (V). The maximum lens resolution was 1,392 (H) × 1,042 (V) with an angle of view of 60°. The camera was placed below an optical waveguide. A heat-resistant borosilicate glass plate was placed between the camera and the waveguide to sustain an optical waveguide without losing the camera resolution. The internal light source was a micro-LED with a diameter of 3 mm. There were four LED light sources placed on four sides of the waveguide to provide sufficient illumination. The direction and incident angle of light were calibrated to be totally reflected in the waveguide. The imaging principle and optimal light incident angle are discussed in the next section.

### 2.2. Sensing Principle

**Figure 2.**
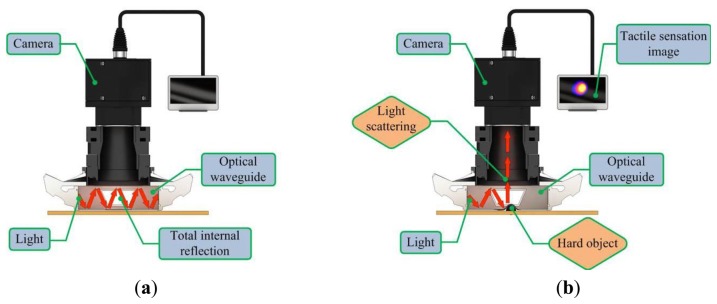
The schematic diagram of the imaging principle. (**a**) The light is injected to the waveguide for total reflection. (**b**) The light scatters as the waveguide deforms according to the external force presented by a hard object.

The proposed sensor operates on the principle of total internal reflection (TIR). According to Snell's law, if two mediums have different refraction indices, and light is shone throughout these two mediums, then a fraction of the light is transmitted and the rest is reflected [20]. If the incident angle is above the critical angle, then TIR occurs. In the current system design, since the waveguide is surrounded by air and has a lower refractive index than the PDMS layers, the incident light directed into the waveguide can be totally reflected in the waveguide. The waveguide is soft and elastic; therefore, if it is compressed by an external force presented by a hard object, the contact area of the waveguide deforms and causes the light to scatter. The scattered light is then captured by the high-resolution camera and saved in image format. The basic principle of tactile sensation imaging lies in the monitoring of light scattering caused by an external force that changes the critical angle. Figure 2 gives a conceptual diagram of the imaging principle.

## 3. Optical Analysis of the Sensing Principle

To investigate the imaging principle, we performed optical analysis. The optical analysis for the one layer waveguide case was carried out using optics communication. In this paper, we extend the one-layer waveguide to a four-layer waveguide. First, the analytical solution for the imaging principle in the four layer waveguide is discussed. Then, the numerical simulation results of our analytical modeling are given.

### 3.1. Analytical Solution

**Figure 3.**
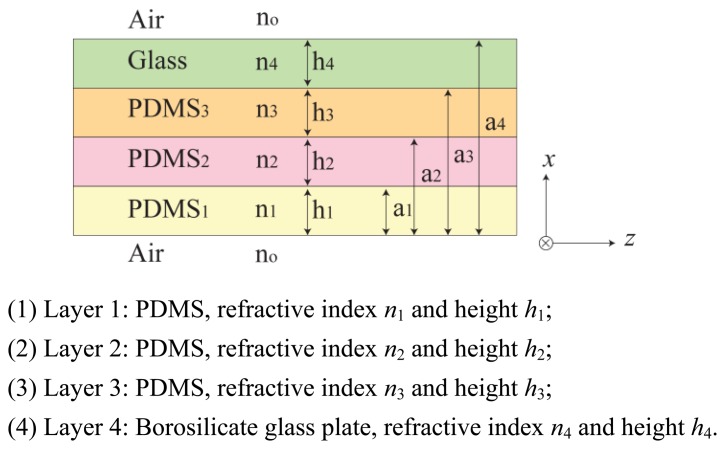
Structure of the multilayer optical waveguide.

In this section, we investigate the imaging principle using the wave optics analysis method. Figure 3 represents an optical waveguide consisting of three PDMS layers with one glass plate layer on top. The refractive index *n*_0_, *n*_5_ is that of the medium surrounding the waveguide, in this case air refractive index, *n*_0_ = *n*_5_ = 1. The layers are positioned in the order of increasing refractive index, *n*_1_ > *n*_2_ > *n*_3_ > *n*_4_ > *n*_0_ = *n*_5_. Light propagates in *z*-direction, and the layers are positioned in *x*-direction. We assume an infinite dimension in the planar *y*-direction.

Let us begin with the Maxwell wave equation describing light propagation in an optical waveguide [10]:

(1) $$\nabla^{2}E(x,y,z,t)-[n^{2}/c^{2}]\partial^{2}E(x,y,z,t)/\partial t^{2}=0$$

Here **E**(*x*, *y*, *z*, *t*) is the electric field, *n* is the refractive index, and *c* is the speed of light in a vacuum. A similar equation is valid for the magnetic field **H**(*x*, *y*, *z*, *t*):

(2) $$\nabla^{2}H(x,y,z,t)-[n^{2}/c^{2}]\partial^{2}H(x,y,z,t)/\partial t^{2}=0$$

Since the components of electric and magnetic fields can generally be determined from one another, we will only focus on the electric field. For monochromatic waves with frequency ω, the solution for Equation (1) has the following form:

(3) E(x,y,z,t)=E(x,y,z)exp(−iωt)

Using the form given in Equation (3) in Equation (1), the spatial distribution of electric field **E**(*x*, *y*, *z*) can be given form [10]:

(4) $$\frac{\partial^{2}E}{\partial x^{2}}+\frac{\partial^{2}E}{\partial y^{2}}+\frac{\partial^{2}E}{\partial z^{2}}+k_{0}^{2}n^{2}E=0$$

where *k*_0_ is the wave vector in a vacuum, *k*_0_ = ω/*c*. Since an optical waveguide is completely uniform in the *z*-direction, we can look only for plane wave solutions:

(5) E(x,y,z)=E(x,y)exp(−iβz)

where *β* is the propagation constant. This is analogous to free wave propagation in form of a plane wave in a bulk medium. Since we are only looking for plane wave solutions, which are independent of the *y*-direction, the field distribution varies only across *x*-dimension:

(6) E(x,y)=E(x)

Further, let us first consider the solution for the transverse *y*-component of the electric field. For this purpose, let us assume that:

(7) E(x)=e(x)j

where **j** is the unit vector along the *y*-direction. By substituting Equation (8) into Equation (4), we can reduce it to the following ordinary differential equation:

(8) $${\text{d}}^{2}e(x)/\text{d}x^{2}+[{k_{0}}^{2}n^{2}-\beta^{2}]e(x)=0$$

This equation has to hold in all optical waveguide layers as well as in air:

(9) $$\begin{array}{cc} {\text{d}}^{2}e(x)/\text{d}x^{2}+[{k_{0}}^{2}{n_{0}}^{2}-\beta^{2}]e(x)=0, & \text{if}\;x<0 \end{array}$$

(10) $$\begin{array}{cc} {\text{d}}^{2}e(x)/\text{d}x^{2}+[{k_{1}}^{2}{n_{1}}^{2}-\beta^{2}]e(x)=0, & \text{if}\;0<x<a_{1} \end{array}$$

(11) $$\begin{array}{cc} {\text{d}}^{2}e(x)/\text{d}x^{2}+[{k_{2}}^{2}{n_{2}}^{2}-\beta^{2}]e(x)=0, & \text{if}\;a_{1}<x<a_{2} \end{array}$$

(12) $$\begin{array}{cc} {\text{d}}^{2}e(x)/\text{d}x^{2}+[{k_{3}}^{2}{n_{3}}^{2}-\beta^{2}]e(x)=0, & \text{if}\;a_{2}<x<a_{3} \end{array}$$

(13) $$\begin{array}{cc} {\text{d}}^{2}e(x)/\text{d}x^{2}+[{k_{4}}^{2}{n_{4}}^{2}-\beta^{2}]e(x)=0, & \text{if}\;a_{3}<x<a_{4} \end{array}$$

(14) $$\begin{array}{cc} {\text{d}}^{2}e(x)/\text{d}x^{2}+[{k_{5}}^{2}{n_{5}}^{2}-\beta^{2}]e(x)=0, & \text{if}\;x>a_{4} \end{array}$$

Since we are interested in solutions that are guided in all four layers and are evanescent outside of the waveguide, let us look for solutions of Equations (10) to (15) in the following form:

(15) $$\begin{array}{cc} e=e_{0}\exp\left[\kappa_{0}x\right], & \text{if}\;x<0 \end{array}$$

(16) $$\begin{array}{cc} e=e_{1}\cos\left[\kappa_{1}x+\varphi_{1}\right], & \text{if}\;0<x<a_{1} \end{array}$$

(17) $$\begin{array}{cc} e=e_{2}\cos\left[\kappa_{2}x+\varphi_{2}\right], & \text{if}\;a_{1}<x<a_{2} \end{array}$$

(18) $$\begin{array}{cc} e=e_{3}\cos\left[\kappa_{3}x+\varphi_{3}\right], & \text{if}\;a_{2}<x<a_{3} \end{array}$$

(19) $$\begin{array}{cc} e=e_{4}\cos\left[\kappa_{4}x+\varphi_{4}\right], & \text{if}\;a_{3}<x<a_{4} \end{array}$$

(20) $$\begin{array}{cc} e=e_{5}\exp\left[\kappa_{5}(h_{1}+h_{2}+h_{3}+h_{4}-x)\right], & \text{if}\;x>a_{4} \end{array}$$

As no light propagates outside the waveguide, the assumed solution in regions x < 0 and x > a_4_ must decay exponentially with the distance from the surface. Meanwhile, the propagating lights in regions 0 < x < a_1_, a_1_ < x < a_2_, a_2_ < x < a_3_, and a_3_ < x < a_4_ are oscillating and have sinusoidal form. The solutions are determined with unknown parameters such as amplitudes ei, transverse wave vectors κi, and phases φi, i = 1,2,3,4. These parameters will have to be determined from the boundary conditions, matching the fields in different regions. Intensity of the LED light is the square of e: I = | e |2.

### 3.2. Numerical Simulation

**Figure 4.**
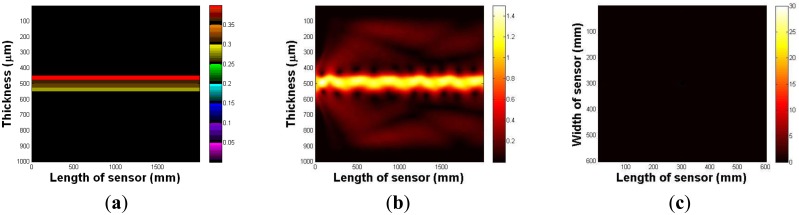
(**a**) The optical waveguide, as seen from the side. (**b**) The light oscillation in the optical waveguide due to the Snell's law. (**c**) The captured image from the top surface of the optical waveguide.

In this section, the imaging principle is numerically simulated using an analytical solution. Throughout the numerical simulation, we demonstrated the total internal reflection in the multilayer optical waveguide. We also show that if an optical waveguide is deformed by an external force, the light is scattered and seen from the surface of an optical waveguide. Figure 4a represents an optical waveguide before the light injection, as seen from the side. The three PDMS layers and one glass plate layer are represented in different colors. We assume that the light is injected from the left side of the waveguide. The light injection result is shown in Figure 4b. Once the light is injected into the waveguide, a small portion of light diffracts away because of the discontinuity of the mediums, air, and the waveguide. However, due to Snell's law, we can clearly see the sinusoidal oscillation of the other light, and it continues to propagate in the waveguide. We have also simulated the tactile sensation image. We captured any scattering light from the top surface of the optical waveguide; Figure 4c shows the result. We can verify that since the light is completely reflected in the optical waveguide, and there is no captured scattering light.

**Figure 5.**
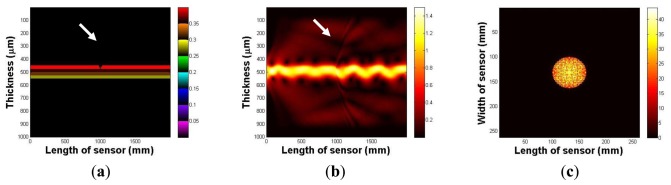
(**a**) The optical waveguide, as seen from the side. (**b**) Light scattering in the optical waveguide due to the waveguide deformation. (**c**) Captured image from the top surface of the optical waveguide.

Next, we investigate the light scattering in the case of waveguide deformation. In this simulation, we use the same waveguide, except it has a small deformation, 5 mm depth, and a 10 mm radius on the top surface. The optical waveguide with a small deformation is shown in Figure 5a. Figure 5b shows the light injection result. Once we inject the light into the waveguide, we can clearly see that the light hits the deformed region, which causes the scattering light from the surface of the waveguide. In Figure 5c, we captured scattering light from the waveguide.

### 3.3. Finite Element Analysis of the Sensing Principle

**Figure 6.**
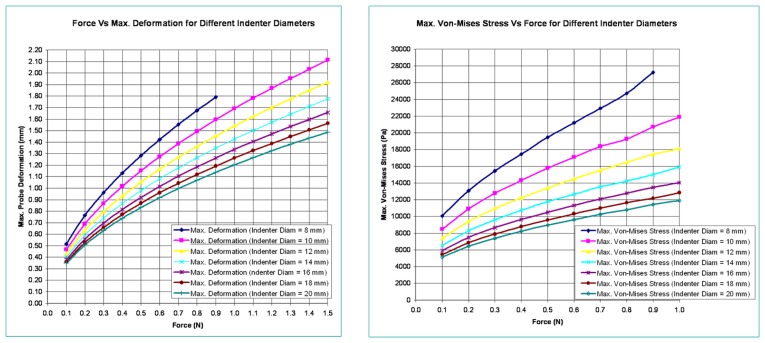
Deformation and Von-Mises stress variation for different indenter diameters and applied normal forces.

In this section, the optical waveguide deformation has been verified using the numerical simulation called finite element analysis (FEA). In FEA, the indenter to press the optical waveguide of the sensor is made of steel and is spherical in shape. Various diameters for the indenter are considered in the analysis. The optical waveguide is made up of three layers. Each has a different thickness and modulus of elasticity. Its cross-section measures 30 mm × 30 mm, and the thickness of all three layers combined is 9 mm. The spherical steel indenter has a modulus of elasticity value of 210 GPa. In this analysis, the optical waveguide of the sensor is pressed with a spherical steel indenter and various results like probe deformation, stress, total deformed area, and surface displacement summation are obtained. The indenter is pressed both in the normal and tangential directions. Figure 6 shows the maximum deformation and Von-Mises stress variations relative to indenter diameters and variations in applied normal forces.

**Figure 7.**
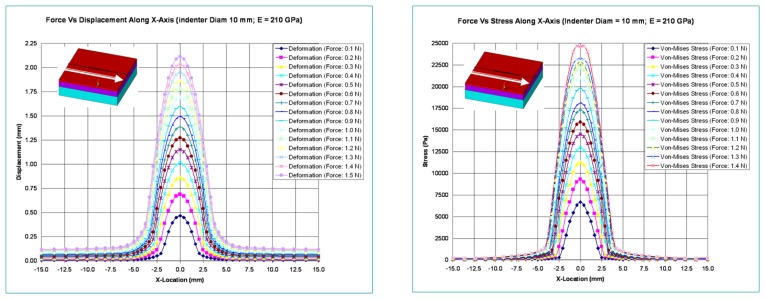
Deformation and Von-Mises stress along the length of the optical waveguide.

Figure 7 shows the variation of deformation and stresses along the length of the optical waveguide for an indenter diameter of 10 mm.

## 4. Experimental Results

In this section, we demonstrate the capability of the sensor to characterize an inclusion in soft tissue. For this experiment, three tissue phantoms with embedded hard inclusions (simulated tumors) were manufactured. The tissue phantoms consisted of size, depth, and hardness tissue phantoms. Each phantom comprised three inclusions. The phantom was made of a silicone composite with a Young's modulus of approximately 5 kPa. The inclusion was generated using another silicone composite, the stiffness of which was higher than the surrounding tissue phantom. The compression level was 500 mN in all the experimental results. The calculation method of size, depth, and Young's modulus and experimental results of a phantom study are given as below. The units of size, depth and hardness are mm, mm, and kPa.

### 4.1. Inclusion Size Estimation

In this paper, we assume that an inclusion is spherical. In the device operation, as the diameter of an inclusion increases, the light scattering increases as the effect of bigger inclusion causes more change in the optical waveguide deformation. Thus, we measured the light scattering area of an image to estimate the inclusion diameter. Let *I*(*x,y*) be the individual pixel value of an image. Then the light scattering area *A* captured in the image can be calculated by counting the number of pixels bigger than the specific value of *k*:

(21) A=number of pixel values

that is the number of *I* (*x,y*) ≥ *k k. k* is the pixel threshold value and we set it as 5. Then a relative diameter size of an inclusion *d* can be found as follows:

(22) $$d=2\sqrt{A/\pi }.$$

The diameter size of Equation (22) is the pixel distance in the image. To transform the pixel distance to the real distance, the scale factor is used. The scale factor between the actual distance and the image pixel distance is 6.79 × 10^−3^ mm per pixel. We obtained this ratio by the calibration. The unit of the relative inclusion diameter size is mm.

### 4.2. Inclusion Depth Estimation

As the depth of inclusion increases, the light scattering due to the waveguide deformation decreases as the effect of a inclusion becomes reduced. Since we have assumed that the inclusion is spherical, the pixel values of the tactile sensation image distribute in a bell shape, where the pixel intensity is the highest at the centroid of an image and decreases with increasing distance from the centroid. Thus, we used a centroid pixel value of an image to estimate a relative inclusion depth. The *x*- and *y*-coordinates of the image centroid, (*X_c_,Y_c_*), are calculated by:

(23) $$X_{c}=\frac{\iint_{x,y}I(x,y)\text{xdxdy}I(x,y)\text{xdxdy}}{\iint_{x,y}I(x,y)\text{dxdy}I(x,y)\text{dxdy}}$$

(24) $$Y_{c}=\frac{\iint_{x,y}I(x,y)y\text{dxdy}I(x,y)y\text{dxdy}}{\iint_{x,y}I(x,y)\text{dxdy}I(x,y)\text{dxdy}}$$

Then a relative depth of an inclusion *h* can be calculated as below:

(25) $$h=I(X_{c},Y_{c})$$

Same as the inclusion size estimation case, the same scale factor is used to transform the pixel distance to the actual distance. Thus, the unit of the relative inclusion depth is mm.

### 4.3. Inclusion Hardness Estimation

The word “hardness” is expressed by Young's modulus *E*. Young's modulus is expressed as stress over strain as below:

(26) E=stress/strain

The stress is measured as force per unit area. In this paper, we estimate the force *F* using the integrated pixel value *M* of the tactile sensation image. The relationship between the force *F* and the integrated pixel value *M* is obtained from the initialization:

(27) $$F=1.0\times 10^{6}\times (0.056+1.478M)$$

where:

(28) $$M=\iint_{x,y}I(x,y)\text{dxdy}I(x,y)\text{dxdy}.$$

Since the stress is measured as force per unit area, the final estimated stress, *P*, is as follows:

(29) P=F/C

where *C* is the bottom surface area of the optical waveguide.

The other value needed for the elastic modulus is the strain. The strain is the fraction change in length in response to the stress. Strain is the geometrical deformation measure indicating the relative displacement between points on the target. Thus, if we know the displacement of any particular set of points on tactile sensation image obtained under different loading forces to the target, then we can find the strain presented by the loading forces. To find the stain *T*, first we obtained two tactile sensation images under different compression ratios. Then we measured the relative diameter using Equation (22). The estimate strain is measured by the difference of the each relative diameter as below:

(30) $$T=d_{1}-d_{2}.$$

The obtained stress and strain are finally used to estimate the relative hardness of an inclusion. The unit of the relative hardness of an inclusion is Pa.

### 4.4. Size Phantom Experiment

**Figure 8.**
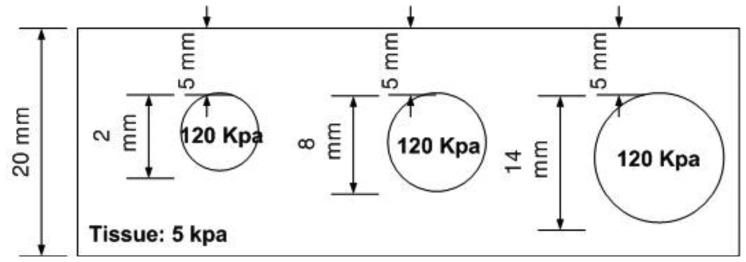
Schematic of the size phantom.

**Figure 9.**
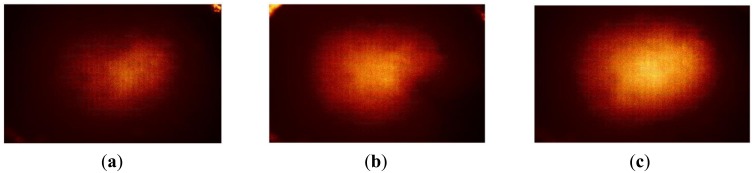
The tactile sensation image of three inclusions embedded in the size phantom: (**a**) 2 mm diameter inclusion, (**b**) 8 mm diameter inclusion, (**c**) 14 mm diameter inclusion.

**Figure 10.**
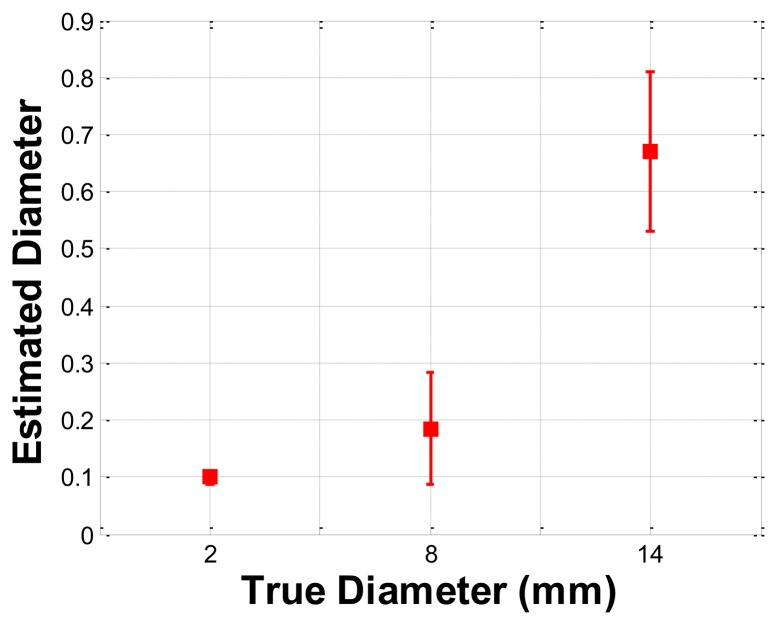
Error bar chart of the estimated relative diameter size of each inclusion.

The size phantom includes three hard inclusions with diameter sizes 2 mm, 8 mm, and 14 mm. Each inclusion was placed 5 mm below the surface of the phantom. The Young's modulus of each inclusion was 120 kPa. The schematic of the size phantom is shown in Figure 8. For each inclusion case, we obtained five tactile sensation images, and then the relative diameter of an inclusion was estimated and averaged. Figure 9 represents the sample tactile sensation images of each inclusion. For better visualization, the image has been normalized. The diameter size estimation results are shown in Figure 10. The plot shows that when the diameter size was 14 mm, the highest mean diameter size of 0.6697 mm was obtained; this was also the most variable, with a standard deviation of 0.2416 mm. Conversely, in the case of a diameter size of 2 mm, the lowest mean diameter size was obtained, at 0.0998 mm; this was also the least variable, with a standard deviation of 0.0197 mm. Since we estimated the relative value, the comparison ratio of each diameter size is also important. The ratio of the real diameter size of the inclusions was approximately 1 mm:4 mm:7 mm. The estimated ratio of relative diameter size was approximately 1 mm:1.85 mm:6.71 mm.

### 4.5. Depth Phantom Experiment

**Figure 11.**
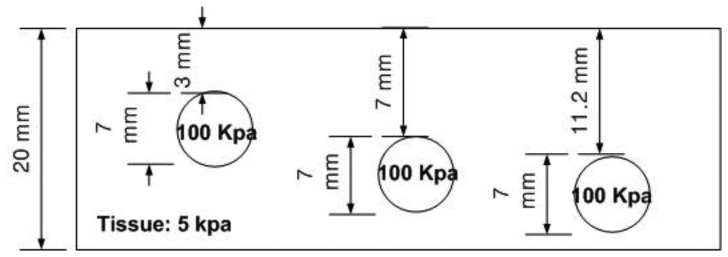
Schematic of the depth phantom.

**Figure 12.**
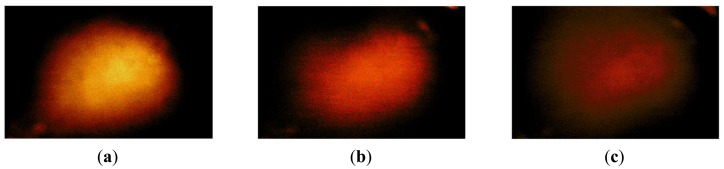
Tactile sensation images of three inclusions embedded in the depth phantom: (**a**) 3 mm depth inclusion, (**b**) 7 mm depth inclusion, (**c**) 11.2 mm depth inclusion.

**Figure 13.**
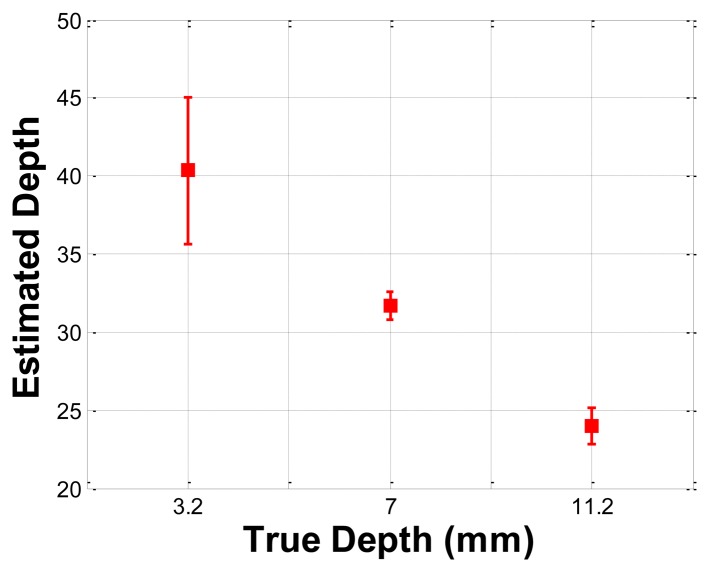
Error bar chart of the estimated relative depth of each inclusion.

The depth phantom had three inclusions with different depths, specifically 3 mm, 7 mm, and 11.2 mm. The diameter size of every inclusion was 7 mm and the inclusions' Young's modulus was 100 kPa. A schematic of the depth phantom is shown in Figure 11. Consistent with the previous size phantom case, we obtained five tactile sensation images for each inclusion. Then, the relative depth of an inclusion was estimated and averaged. Figure 12 represents the sample tactile sensation images of each inclusion. The depth estimation result is shown in Figure 13. The plot shows that the 3 mm depth case had the highest mean of 40.33 mm, and the most variable standard deviation at 8.08 mm, while the 11.2 mm depth case had the lowest mean depth of 24 mm, and 7 mm depth case had the least variable, at 1.52 mm. The ratio of the real depth of inclusions was approximately 1 mm:2.3 mm:3.7 mm. The estimated relative depth ratio was approximately 1 mm:0.79 mm:0.59 mm.

### 4.6. Hardness Phantom Experiment

**Figure 14.**
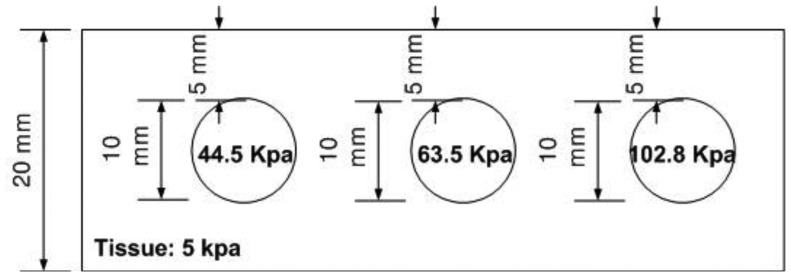
Schematic of the hardness phantom.

**Figure 15.**
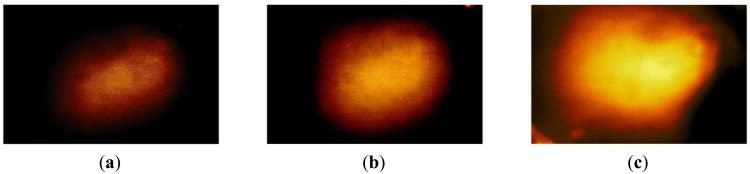
Tactile sensation images of three inclusions embedded in the hardness phantom: (**a**) 44.5 kPa Young's modulus inclusion, (**b**) 63.5 kPa Young's modulus inclusion, (**c**) 102.8 kPa Young's modulus inclusion.

**Figure 16.**
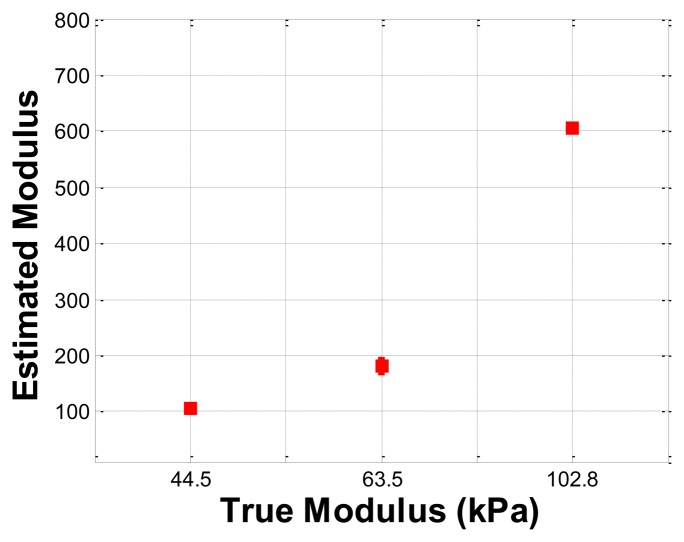
Error bar chart of estimated relative Young's modulus of each inclusion.

The hardness phantom had three inclusions with different Young's moduli, at 44.5 kPa, 63.5 kPa, and 102.8 kPa. The diameter size of inclusions was 10 mm, and they were placed 5 mm below the surface of the phantom. The schematic of the depth phantom is shown in Figure 14. Using multiple compression ratios, we obtained 10 tactile images of each inclusion. Then, the relative Young's modulus of an inclusion was estimated and averaged. Figure 15 represents the sample tactile sensation images of each inclusion. The Young's modulus estimation result is shown in Figure 16.

The plot shows that the 102.8 kPa Young's modulus case had the highest mean of 605.2 kPa, and the smallest standard deviation, indicating that the observations were close to the mean. In contrast, the 63.5 kPa Young's modulus case had the most widely spread out Young's modulus, as can be seen in its error bar chart. The 44.5 kPa Young's modulus had an average of 105.9 kPa, and a standard deviation of 6.66 kPa. The ratio of the real Young's modulus of the inclusions was approximately 1 kPa:1.43 kPa:2.31 kPa. The estimated relative Young's modulus was approximately 1 kPa:1.71 kPa:5.71 kPa.

## 5. Conclusions

In this paper, an optical-based, artificial palpation sensor for lesion characterization capable of characterizing the inclusions in tissue was designed and experimentally evaluated. To emulate the human finger layer, a multilayer optical waveguide was fabricated as the main sensing probe. The total internal reflection principle was used to obtain the high resolution tactile image. In order to obtain relative size, depth, and Young's modulus of an inclusion, a new image processing algorithm was proposed. The performance of the sensor was experimentally verified using tissue phantoms with embedded hard inclusions. The proposed tactile sensation imaging system (TSIS) is a hand held probe and it is manipulated by hand, thus, the control of the indentation of the TSIS in different areas is very important. In the paper, we control the TSIS indentation using the external machine such as the loading machine with pressure gauge to investigate the relationship between FEM tactile data and TSIS tactile data in the idealized conditions. For the clinical experiments, however, the data were collected by hand-held, not mechanically assisted. The experimental findings of the measurement performance under the hand-held condition and idealized conditions will be evaluated as the future work. In this paper, the size, depth, and hardness have been determined with one single image. Figure 10, Figure 13, and Figure 16 are the relative value composites. To find the absolute values of size, depth, and hardness, we need at least two images. In future work, absolute value calculation schemes will be investigated using two different images. This work is the initial step towards achieving a tactile sensation imaging system for embedded breast tumor detection and characterization.
